# Green Synthesis and Characterization of Silver Nanoparticles Using *Myrsine africana* Leaf Extract for Their Antibacterial, Antioxidant and Phytotoxic Activities

**DOI:** 10.3390/molecules27217612

**Published:** 2022-11-06

**Authors:** Qudsia Sarwer, Muhammad Shoaib Amjad, Ansar Mehmood, Zakia Binish, Ghazala Mustafa, Atikah Farooq, Mirza Faisal Qaseem, Fozia Abasi, José Manuel Pérez de la Lastra

**Affiliations:** 1 Department of Botany, Women University of Azad Jammu & Kashmir, Bagh 12500, Pakistan; qudsiasarwar098@gmail.com (Q.S.); zakiabenish88@gmail.com (Z.B.); 2 School of Geography, Earth and Environmental Sciences, University of Birmingham, Birmingham B15 2TT, UK; 3 Department of Botany, University of Poonch, Rawlakot 12350, Pakistan; ansar.mehmood321@gmail.com; 4 Department of Plant Sciences, Quaid-i-Azam University, Islamabad 45320, Pakistan; ghazala.mustafa@yahoo.com (G.M.); atikahfarooq@bs.qau.edu (A.F.); 5 Department of Environmental Science and Forestry, Connecticut Agricultural Experiment Station, 123 Huntington Street, New Haven, CT 06511, USA; faisal.ali522@gmail.com; 6 Department of Botany, PMAS-University of Arid Agriculture, Rawalpindi 44000, Pakistan; abasifozia@gmail.com; 7 Biotecnología de Macromoléculas, Instituto de Productos Naturales y Agrobiología, (IPNA-CSIC), 38206 San Cristóbal de la Laguna, Spain

**Keywords:** AgNPs, biosynthesized, *Myrsine africana*, biological activities

## Abstract

Nanotechnology is the study and control of materials at length scales between 1 and 100 nanometers (nm), where incredible phenomena enable new applications. It affects all aspects of human life and is the most active research topic in modern materials science. Among the various metallic nanoparticles used in biomedical applications, silver nanoparticles (AgNPs) are among the most important and interesting nanomaterials. The aim of this study was to synthesize AgNPs from the leaf extract of *Myrsine africana* to investigate their antibacterial, antioxidant, and phytotoxic activities. When the leaf extract was treated with AgNO_3_, the color of the reaction solution changed from light brown to dark brown, indicating the formation of AgNPs. The UV-visible spectrum showed an absorption peak at 438 nm, confirming the synthesis of AgNPs. Scanning electron microscopy (SEM) showed that the AgNPs were spherical and oval with an average size of 28.32 nm. Fourier transform infrared spectroscopy confirms the presence of bio-compound functional groups on the surface of the AgNPs. The crystalline nature of the AgNPs was confirmed by XRD pattern. These biosynthesized AgNPs showed pronounced antibacterial activity against Gram-positive and Gram-negative bacteria, with higher inhibitory activity against *Escherichia coli*. At 40 µg/mL AgNPs, the highest antioxidant activity was obtained, which was 57.7% and an IC50 value of 77.56 µg/mL. A significant positive effect was observed on all morphological parameters when AgNPs were applied to wheat seedlings under constant external conditions at the different concentrations. The present study provides a cost-effective and environmentally friendly method for the synthesis of AgNPs, which can be effectively used in the field of therapeutics, as antimicrobial and diagnostic agents, and as plant growth promoters.

## 1. Introduction

Recently, nanotechnology has attracted the attention of researchers due to its wide range of applications in the fields of medicine, agriculture, environment, and food [1]. This technology essentially focuses on the synthesis of tiny nanoparticles produced by chemical, physical, and biological processes, which contribute significantly to the control of plant and animal diseases and have shown considerable promise in improving the quality of human life and health conditions [2]. These nanoparticles range in size from 1 to 100 nm. However, the green synthesis of nanoparticles has become more important than chemical and physical methods [3]. Green synthesis involves the use of plants for the synthesis of various types of nanoparticles [4]. Green synthesis is a simple, environmentally friendly, non-polluting, antitoxic, and cost-effective technique. Green synthesized metal nanoparticles are the most innovative and promising agents for a variety of biological and catalytic activities, such as antibacterial, antiviral, anticancer, etc., without negative side effects [5]. In green synthesis, plant extracts are used as capping agents and important stabilizers that control the growth of nanoparticles and prevent them from aggregating or coagulating. These stabilizing substances are essential for altering biological processes and environmental perception [6]. 

Common risk factors pathologically associated with various human diseases include infection, oxidative stress, angiogenesis, and inflammation. During infection, pathogenic microorganisms invade the host body and the host tissues respond to the spread of this infection. Bacterial infections have become a global public health problem, resulting in millions of infection-related illnesses and deaths each year [7]. Typical bacterial infectious diseases include keratitis, histoplasmosis, tuberculosis, as well as dental and skin infection [8]. The effectiveness of current antibiotics is being eroded by pathogen resistance [9], which is now a problem for everyone’s health. Due to the rapid increase in antibiotic resistance, scientists and researchers are currently investigating the therapeutic potential of silver and its nanoparticulate systems as potential antibacterial agents [10]. Currently, silver nanoparticles (AgNPs) have shown their potential as alternative antibacterial agents in many studies [11,12,13]. AgNPs have been found to exhibit antiseptic activity against several bacterial species, including multidrug-resistant bacteria such as methicillin-resistant bacteria, and are safe for mammalian cells at low concentrations [14]. Oxidative stress refers to the overproduction of reactive oxygen species (ROS). These ROS react with various biological molecules, such as DNA, protein, and lipid membranes, thereby impairing inter or intra cellular function by inducing cellular damage which leads to different diseases [2] such as diabetes [15], arthritis [16], cancers [17], Alzheimer’s disease [18], and cardiovascular disease [19]. Various studies have confirmed that green synthesized silver nanoparticles have stronger antioxidant activity due to the presence of various biomolecules on their surface and can be used as radical scavengers against the damage caused by free radicals [2,20,21,22]. Some of the biological uses of AgNPs include antioxidant, antibacterial, anti-inflammatory, wound healing, anticancer, antiproliferative, antifungal, antiviral, and antidiabetic properties [23]. Because of these unique properties, silver nanoparticles (AgNPs) are extensively used for drug delivery, therapeutic devices, sensing and diagnostics, and other applications. AgNPs are of critical importance due to their catalytic activity, optical, and thermal properties, chemical stability, thermal stability, and antibacterial activity [24].

In this study, AgNPs were synthesized from the leaf extract of *Myrsine africana* L. (*Primulaceae*). The shrub *M. africana* is better known as African boxwood. It is widely distributed in Asia, the Caribbean and Africa, and a wealth of traditional knowledge relates to its use. Traditionally, its leaves are used for the treatment of cellulitis, acne, wound healing and pigmentation disorders [25]. This is the first report on the synthesis of AgNPs from the leaf extract of *M. africana*, although the environmentally friendly production of AgNPs for use in biomedicine has been thoroughly investigated with a variety of plant extracts [26]. Therefore, the following objectives were pursued in this study: (i) green synthesis and characterization of AgNPs from the leaf extract of *M. africana*; and (ii) evaluation of the antibacterial, antioxidant, and phytotoxic activities of AgNPs.

## 2. Material and Methods

### 2.1. Collection of Sample and Preparation of Plant Extract

The leaves of *M. africana* were collected by Qudsia Sarwer from Kahutta Azad Jammu and Kashmir in April 2021. The plant was identified by Dr. Muhammad Shoaib Amjad with the help of Flora of Pakistan and a voucher specimen (voucher number 278) was deposited in the Herbarium of the Department of Botany, Women University of Azad Jammu and Kashmir Bagh. The fresh leaves were washed under running tap water to remove impurities and dust particles and then dried in shade to prepare the powder. To prepare the leaf extract, 10 g of powder was placed in a 250 mL conical flask containing 100 mL of distilled water and heated on a magnetic stirrer at 60 °C for 1 h. It was then filtered with Whatman No. 1 filter paper and the leaf filtrate was used for the synthesis of AgNPs [27].

### 2.2. Green Synthesis of AgNPs

To prepare the silver nitrate stock solution (1 mM), 80 mL of the plant leaf extract was mixed with 250 mL of the silver nitrate stock solution and incubated for 3 h at room temperature. After 3 h of incubation, a visual color change of the reaction solution was observed [27].

### 2.3. Characterization of Silver Nanoparticles

#### 2.3.1. UV-Visible Spectroscopy

To determine the absorbance value of the nanoparticles in the range of 300–600 nm, a UV-Vis spectrophotometric study was performed [28]. The UV-Vis spectrophotometer was used to record the SPR of the biogenic AgNPs [29].

#### 2.3.2. SEM-EDX 

Scanning electron microscopy coupled with energy-dispersive X-rays (SEM-EDX) was used to analyze the morphology and composition of Ag-NPs [30]. A histogram of the size distribution of AgNPs was created using imageJ software. 

#### 2.3.3. XRD

The crystalline structure of the biosynthesized Ag-NPs was investigated by X-ray diffraction (XRD). The sample was analyzed between 3 and 90 theta values. Using Cu-Ka as the radiation source (=1.54), the XRD investigation was performed at a voltage and current of 40 KV and 30 mA, respectively [31]. 

#### 2.3.4. FT-IR

The different functional groups responsible for the reduction and stabilization of the biosynthesized Ag-NPs were identified using FT-IR analysis (Cary 630 FTIR model, Tokyo, Japan). The KBr technique was used to perform the following FT-IR analysis: Under high pressure, 300 mg of biosynthesized Ag-NPs were thoroughly mixed with KBr to create a slice that was scanned at a wave number between 400 and 4000 cm^−1^ [32].

### 2.4. Biological Activities

#### 2.4.1. Antibacterial Activity

Four putative bacterial pathogens, *Pseudomonas aeruginosa*, *Escherichia coli*, *Staphylococcus aurous* and *Klebsiella pneumoniae*, were used for antibacterial activity.

The agar well diffusion method was used to evaluate the bactericidal activity of *Myrisine africana* AgNPs. For the bacterial culture, nutrient broth media (Oxide: CM1) was utilized, and nutrient agar (Oxide: CMO337) was used for the bactericidal activity. The overnight culture was combined with freshly made nutrient agar medium and placed into sterile petri plates at 45 °C. For solidification, all petri dishes were placed in a laminar flow at room temperature. The striking of bacterial strains was performed in each plate then different concentrations (0.03, 0.05, 0.09, 0.11, and 0.13 mg/mL) of AgNPs was used and 100 uL of each concentration was put into respective well and was kept at 37 °C for 24 h. The diameter of the inhibition zone after 24 h was measured in millimeters (mm) to determine microbial growth. With the help of scale, the diameter of the clear zones around each well was measured [20,33].

#### 2.4.2. Antioxidant Assay

The DPPH assay was used to determine the antioxidant potential of Ma-extract and Ma- AgNPs [20,34]. The stock solution was prepared by dissolving DPPH (12.5 mg) in 50 mL of methanol, and kept in the refrigerator. The plant sample solution was also prepared in methanol. The serial dilution of the solution was also prepared with different concentration (100, 50, 25, and 12.5 µg/mL). Further, 0.1 mL of each dilution was mixed with 3.0 mL of DPPH in a test tubes in kept them in to the incubator for half an hour at 37 °C. Ascorbic acid was used as standard. UV-Vis spectrophotometer was used to determine the absorbance against the standard at 517 nm. All the test tubes were repeated 3 times for the absorbance. The control sample was also prepared by using 2 mL of DPPH solution + 1 mL of methanol. The result Percentage inhibition was calculated by using given formula.

$$Percentage\;inhibition=\frac{Absorbance\;of\;test}{Absorsbance\;of\;control}\times 100$$

### 2.5. Phytotoxicity Assesement of AgNPs

To study the phytotoxicity of AgNPs, morphological parameters of wheat cultivar Bakhtawar-38 were evaluated under the application of AgNPs. Healthy seeds collected from the National Agriculture Research Center (NARC) in Islamabad, Pakistan, were surface sterilized with 3% sodium hypochlorite for 5 min and then washed three times with distilled water. Seeds were then sown in sand under optimal conditions (65% humidity, +25 °C temperature, and an 8/16-h dark and light period). Eight-day-old wheat seedlings (two-leaf stage) were treated with AgNPs at concentrations of 10, 20, 40, and 80 mg/L. Plant roots were treated with nanoparticle solutions and the effects on morphology (plant fresh weight, root/shoot fresh weight, and root/shoot length) were recorded on the three consecutive days after treatment (i.e., day 9, 10, and 11). Root/shoot length was measured in cm using a measuring scale, while fresh weight was recorded in mg using an electric balance. The experiment was performed in triplicate.

### 2.6. Statistical Analysis

Data were statistically analyzed using Origin Pro version 9.1 software. All tests were performed in triplicate. Analysis of variance (ANOVA) was performed using the least significant differences (LSD) test to compare the significant differences (*p* ≤ 0.05) between groups. 

## 3. Results and Discussion

### 3.1. Green Synthesis and Characterization of AgNPs

**Figure 1:**
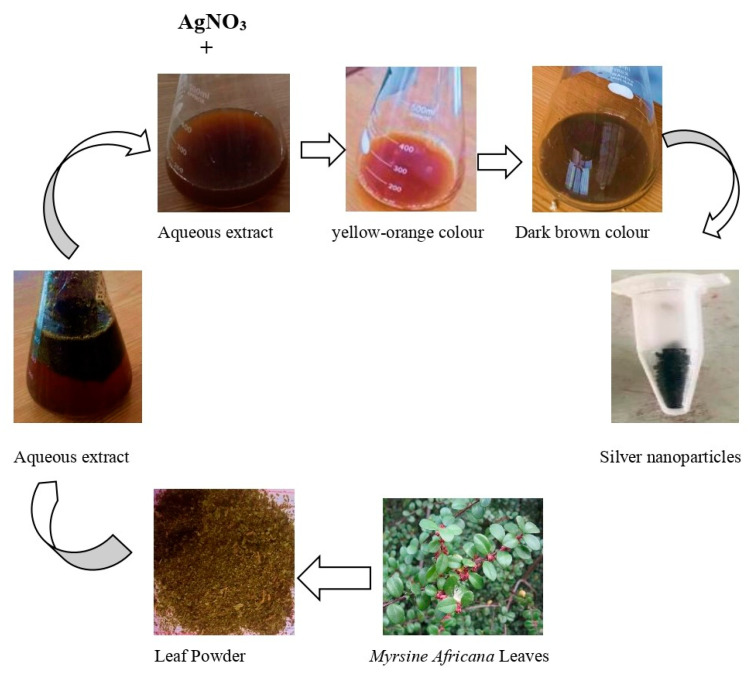
Schematic representation of synthesis of silver nanoparticles using *M. africana* leaf extract. The image of *M. africana* used in this figure can be found on Wikipedia (https://en.wikipedia.org/wiki/Myrsine_africana), last accessed on 17 September 2022.

The plant extract and silver nitrate salt solution were mixed and incubated for 3 h. During this time, there was a visible color change in the reaction mixture. The apparent color of the solution changed from yellow-orange to dark brown, confirming the phytosynthesis of silver nanoparticles (Figure 1). This color change was due to the surface plasmon resonance (SPR) activity of the NPs [35,36]. The amount of color is determined by the number of electrons that are released during the reduction of NO_3_ to NO_2_, which reduces Ag^+^ to metallic ions (Ag^0^) [37]. Our work was supported by the findings of [38], which were identical to ours in terms of the visual change of the color of the solution.

**Figure 2:**
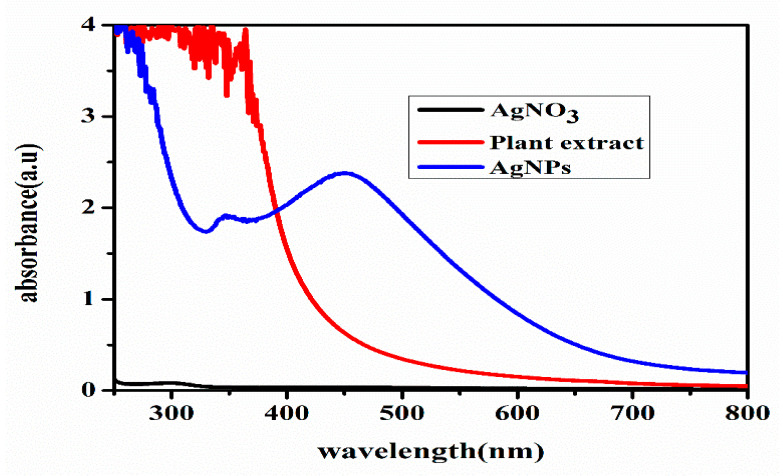
UV. Visible spectroscopy of AgNPs.

The color change of the reaction solution was further investigated using the UV-Vis spectrum of the plant extract and AgNPs. The AgNPs showed an absorption peak at 438 nm caused by SPR due to the excitation of the free electrons of the metal during the formation of the AgNPs (Figure 2). However, the plant extract did not show an absorption peak in this region. In general, these spectra provide information about the properties and formation of colloidal AgNPs. An absorption peak in the range of 400–500 nm is a characteristic feature of AgNPs [39].

**Figure 3:**
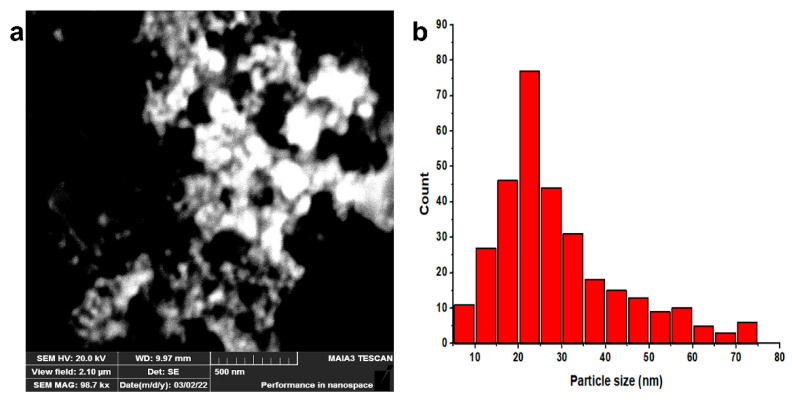
SEM analysis of AgNPs (**a**) SEM (**b**) Particle size distribution.

SEM is a powerful technique for evaluating surface structures, including nanoparticle size, morphology, and shape. A SEM image of these AgNPs shows their spherical and generally uniform shape (Figure 3a). According to the particle size distribution histogram, the average size of AgNPs is 28.32 nm (Figure 3b). Previous studies also support our results [30].

**Figure 4:**
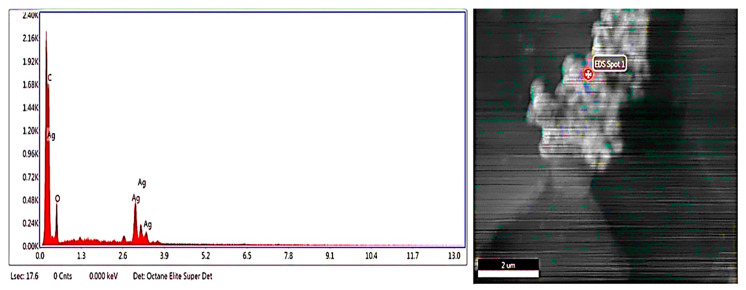
EDX Analysis of AgNPs (**left**) EDX spectrum (**right**) EDX micrograph.

Metallic silver ions were confirmed by energy dispersive X-ray detector. The EDX spectrum showed a strong absorption peak of the metallic silver ions in the range of 2.5–3.7 keV, while the silver nanocrystal showed absorption peaks in the range of 2.5–3.7 keV. Different types of elements in the form of peaks were detected together with silver ions. These elements were carbon and oxygen together with metallic silver (Figure 4).

**Figure 5:**
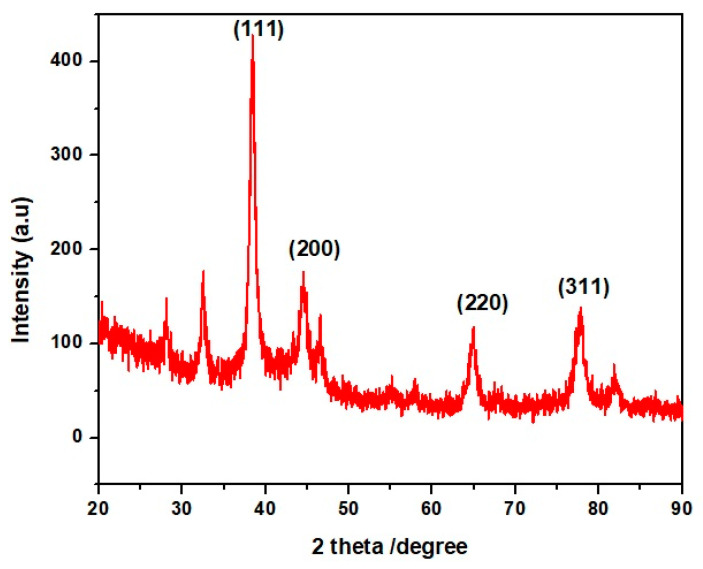
XRD Analysis of AgNPs.

The XRD pattern of AgNPs showed peaks at 38.44, 44.50, 64.80, and 77.94° corresponding to 111, 200, 220, and 311 levels, respectively (Figure 4). These diffraction patterns indicate the crystalline nature of the AgNPs [30]. Additional peaks at 30°, 54°, 56°, and 84 °C were also observed, but these were due to biomolecules in the silver nanoparticles involved in the capping and reduction of AgNPs (Figure 5).

**Figure 6:**
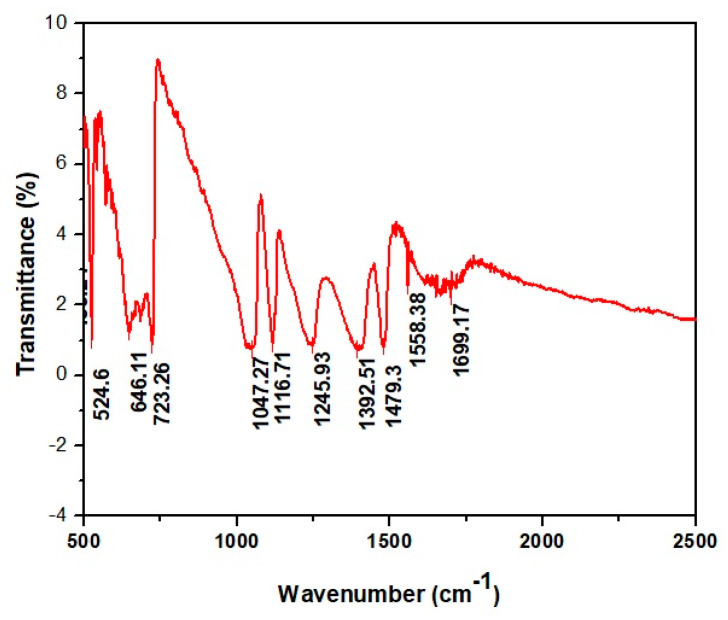
FT-IR Analysis of AgNPs.

FTIR spectroscopy is an essential technique for molecular figure printing, which can detect the functional group of plant secondary metabolites that act as capping or reducing agents [40]. Various bands appeared in the FTIR spectrum between 400 and 4000 cm^−1^. The bands at 1699, 1558, 1479, 1392, 1245, 1116, 1047, 723, 646, and 534 cm^−1^ correspond to C=C, C=O, C-C, C=C, -OH, C-N, and C-X stretching, respectively (Figure 6). These functional groups indicate the presence of plant biocomponents as capping and stabilizing agents of AgNPs. These biomolecules are responsible for the reduction process of Ag^+^ to AgNPs [41].

### 3.2. Biological Activities

#### 3.2.1. Anti-Bacterial Activity

**Figure 7:**
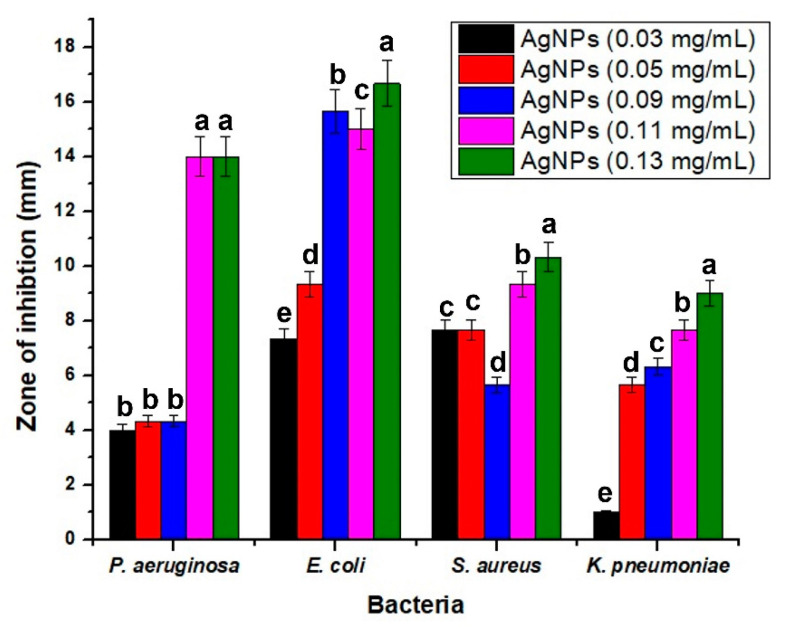
Antibacterial activity of AgNPs at different concentrations against four microorganisms (*P. aeruginosa*, *E. coli*, *S. aureus* and *K. pneumoniae*). All values are the means of 3 replicates, the error bars represent the standard errors of the means, and different letters indicate the significant difference at *p* = 0.05 by LSD test in each group.

The antibacterial activity of *Myrisine africana* AgNPs was evaluated by the agar well diffusion method. Different concentrations of plant based AgNPs were used. When we increased the amount of ANPs, the growth of all bacterial strains was suppressed (Figure 7). All strains showed the greatest inhibition at a dosage of 0.13 mg/mL. The highest antibacterial activity was observed against *E. coli*, a Gram-negative bacterium. It has already been described that even a very low concentration of AgNPs suppresses the growth of bacteria [42]. It is believed that the antibacterial effect of AgNPs is primarily due to the destruction of cell membranes. The antibacterial effect of AgNPs is inhibited by blocking intracellular signaling pathways [43]. In addition, studies revealed that the antibacterial mechanism of biogenic AgNPs is associated with cell membrane disruption, followed by the production of reactive oxygen species, which can alter normal functioning and cause DNA damage and protein denaturation [44]. Furthermore, Ag^+^ ions generated by AgNPs disrupt the bacterial cell cycle, cause mitochondrial dysfunction, and induce apoptosis [45].

#### 3.2.2. Antioxidant Activity

**Figure 8:**
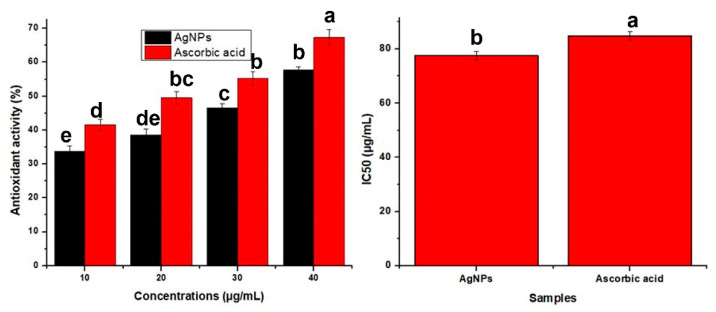
Antioxidant activity of AgNPs. All values are the average of 3 replicates, the error bar represents the standard errors of the means, and different letters indicate the significant difference at *p* = 0.05 by LSD test.

When 2,2-diphenyl-1-picrylhydrazyl accepts an electron or a hydrogen radical, it transforms from a stable synthetic free radical to a stable molecule. Antioxidants convert the DPPH radical to the non-radical form in the DPPH test [46]. As a result, there is less absorption, and the DPPH solution turns yellow instead of purple. The different amounts of phytosynthesized AgNPs (10, 20, 30, 30, and 40 ug/mL) were used and compared with comparable concentrations of ascorbic acid as reference. At 40 ug/mL AgNPs, the highest antioxidant activity was obtained, which was 57.7%, while the standard (ascorbic acid) showed 67.29% at this concentration (Figure 8). Similar results were reported in a previous study [47].

The study of antioxidant activity was conducted because free radicals are the main cause of most human diseases. In the human body, free radicals are the cause of many diseases. Antioxidant monitoring substances control the free radicals produced by our body. Numerous types of antioxidants that are harmful to human health are replaced by natural antioxidants [48]. Antioxidants help reduce oxidative stress in cells and are useful in a variety of human diseases, including inflammation, cardiovascular disease, and cancer [49]. More accessible, bioactive, and safe antioxidants from plants have been explored as an alternative to synthetic antioxidants [50]. AgNPs exhibit potent antioxidant activity by reducing oxidative stress in cells, which is beneficial for the treatment of many diseases such as cancer, lung infections, cardiovascular disease, and inflammatory infections. Previous researchers have studied, contrasted, and verified these findings [51].

## 4. Phytotoxicity of AgNPs

**Figure 9:**
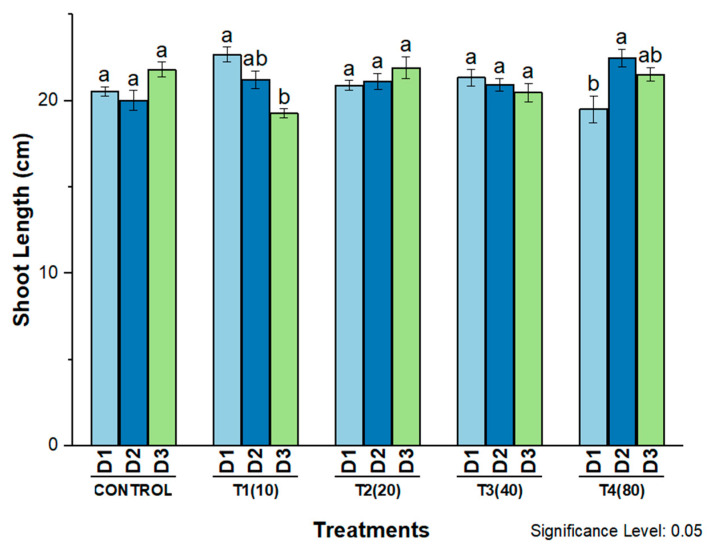
Shoot length of wheat cultivar Bakhtawar-38 in response to 10, 20, 40, and 80 mg/L of AgNPs. All values are the average of 3 replicates, error bars represent the standard errors of the means, and different letters indicate the significant difference at *p* = 0.05 by LSD test in each group.

When AgNPs were applied to wheat seedlings, a significant positive effect was observed for all morphological parameters at the different concentrations under constant external conditions. Shoot length was significantly increased by AgNPs at a concentration of 10 mg/L on days 2 and 3 of treatment and at a concentration of 80 mg/L on days 1 and 3 of treatment. On the other hand, shoot length was increased by 20 mg/L AgNPs and decreased by 40 mg/L AgNPs on each of the first three days after treatment. However, there was no detectable difference between the effect and the control (Figure 9).

**Figure 10:**
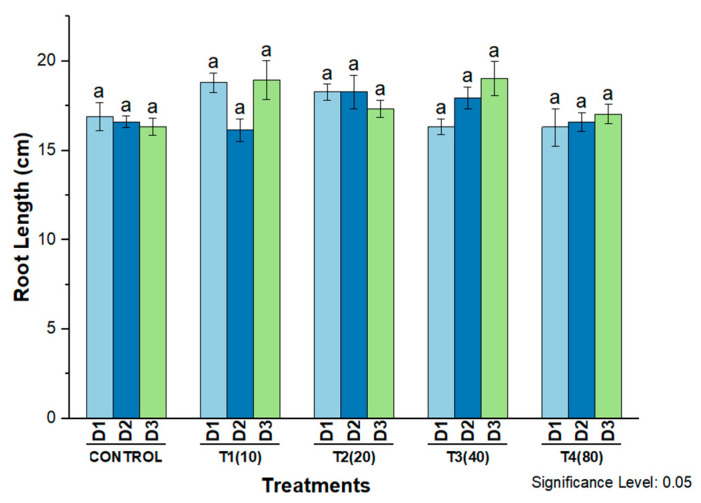
Root length of wheat cultivar Bakhtawar in response to 10, 20, 40, and 80 mg/L of AgNPs. All values are the average of 3 replicates, the error bars represent the standard errors of the means, and different letters indicate the significant difference at *p* = 0.05 by LSD test in each group.

For root length, a favorable effect was observed on 40 mg/L and 80 mg/L AgNPs compared to control plants on all three days after treatment. However, at an exposure level of 20 mg/L, root length initially increased on the first and second days before decreasing on the third day. In response to 10 mg/L AgNPs, root length was significantly increased on days one and three compared to control plants (Figure 10).

**Figure 11:**
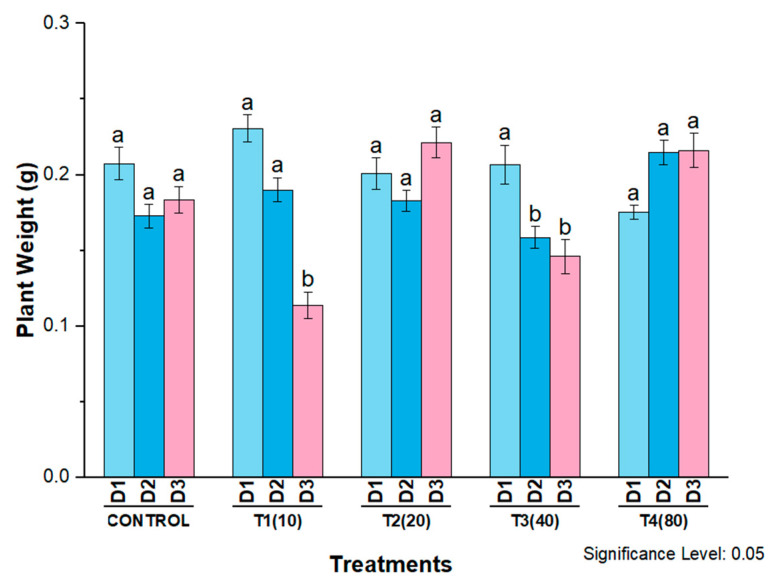
Plant weight of wheat cultivar Bakhtawar-38 in response to 10, 20, 40, and 80 mg/L of AgNPs. All values are the average of 3 replicates, the error bars represent the standard errors of the means, and different letters indicate the significant difference at *p* = 0.05 by LSD test in each group.

The fresh weight of plants was also strongly affected by different concentrations of AgNPs. A significant decrease in seedling fresh weight was observed at the 10 mg/L and 40 mg/L exposure levels compared with the control on all three days after treatment. Control plants and plants treated with AgNPs at 20 mg/L showed a similar trend in fresh biomass. In contrast, only 80 mg/L AgNPs increased plant fresh weight on the second and third days after treatment compared to the other exposure levels and control plants (Figure 11). 

**Figure 12:**
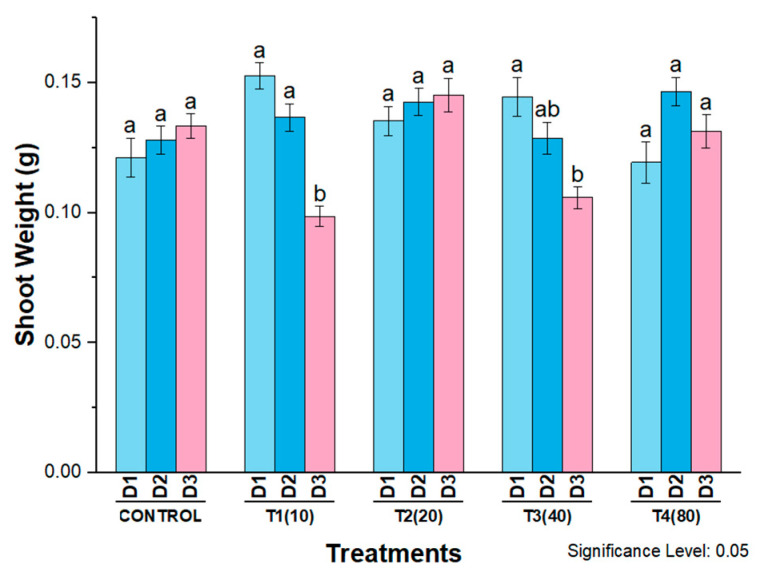
Shoot weight of wheat cultivar Bakhtawar-38 in response to 10, 20, 40, and 80 mg/L of AgNPs. All values are the average of 3 replicates, the error bars represent the standard errors of the means, and different letters indicate the significant difference at *p* = 0.05 by LSD test in each group.

Shoot fresh weight was significantly decreased in response to concentrations of 10 mg/L and 40 mg/L AgNPs on all three days after treatment. The highest increases and decreases in shoot weight were recorded on the first and third days of plants treated with 10 mg/L AgNPs compared with all other treatments and control plants. In contrast, at a concentration of 20 mg/L AgNPs, shoot fresh weight increased continuously on all three days after treatment. In the plants treated with 80 mg/L AgNPs, shoot weight was also increased on the second and third days (Figure 12).

**Figure 13:**
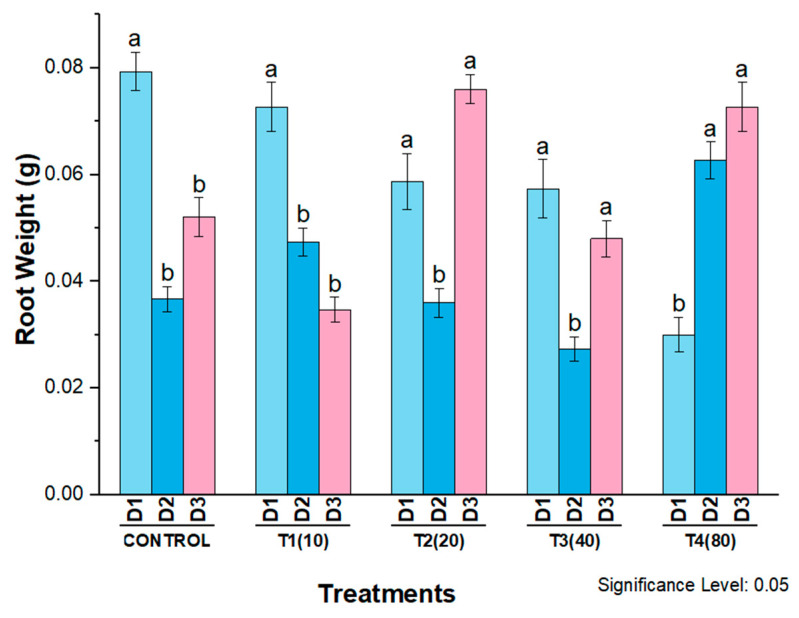
Root weight of wheat cultivar Bakhtawar in response to 10, 20, 40, and 80 mg/L of AgNPs. All values are the average of 3 replicates, the error bars represent the standard errors of the means, and different letters indicate the significant difference at *p* = 0.05 by LSD test in each group.

Different concentrations of AgNPs also showed a significant effect on the fresh weight of roots of eight-day-old wheat seedlings. On the first day, root fresh weight was significantly lower in plants treated with 80 mg/L AgNPs compared to all other treatments on the first day and to the control. On the second day, root weight decreased only in plants treated with 40 mg/L, while it increased in plants treated with 10, 20, and 80 mg/L compared to untreated plants. On the third day, the highest root weight was recorded in the plants treated with 20 mg/L AgNPs compared to the third day of all other treatments and control plants (Figure 13).

Previous studies also reported that AgNPs had a positive effect on plant morphology, including increased fresh weight and longer roots and shoots [20,52]. In our study, the most significant effects were observed on root weight, with all four concentrations showing significant differences compared to the control. Moreover, root weight gradually decreased with increasing concentration on the third day after treatment, suggesting that these effects were dose-dependent. Similar results were obtained by Mirzajani et al. (2013), who found that root growth of *Orzya sativa* L. increased up to 30 g/mL but decreased at 60 g/mL [53]. In our study, a concentration of 10 mg/L AgNPs was found to be non-toxic for all parameters. This finding suggests that lower concentration of AgNPs could be beneficial for plant growth by improving plant growth parameters [54]. Similar results were reported by Mustafa et al., (2020), who found that 10 mg/L AgNPs increased soybean root growth, while 50 mg/L decreased root length and weight [55]. Hasan et al. also reported the same results on lettuce [56]. Our results indicate that 80 mg/L AgNPs at higher concentrations decreased morphological parameters compared with the control, but the effects gradually increased after three days in all treatments. Thus, it appears that the beneficial effects of higher doses are time-dependent. These results are consistent with those of Mustafa et al., (2015, 2016) on soybean, who found that AgNPs at a concentration of 80 mg/L initially increased seedling weight and root length before decreasing under flood conditions [56,57]. Higher concentrations of AgNPs (50 mg/L) also increased hypocotyl weight in a time-dependent manner [55]. This suggests that the phytotoxicity of AgNPs is dose-dependent, while higher concentrations may be time-dependent for their beneficial effects.

## 5. Conclusions

In this study, a sustainable, green approach was adopted for efficient synthesis using the leaf extract of *M. africana*., which yielded spherical shaped AgNPs with an average size of 28.32 nm. *Myrisins africana*-AgNPs showed significant bactericidal activity with a higher zone of inhibition against *E. coli*. They also showed remarkable antioxidant activity in the form of DPPH radical scavenger. Under the different concentrations and constant external conditions, a noticeable positive effect was observed on all morphological parameters of wheat seedlings. The results of the present study suggest that AgNPs can be used as antibiotics, antioxidants, and phytotoxic agents in the future as they are non-toxic, inexpensive, and highly effective.

## Data Availability

Not applicable.

## Sample Availability

Samples of the compounds are not available from the authors.

## References

[1] Pandit C., Roy A., Ghotekar S., Khusro A., Islam M.N., Emran T.B., Lam S.E., Khandaker M.U., Bradley D.A. (2022). Biological agents for synthesis of nanoparticles and their applications. J. King Saud Univ. Sci..

[2] Nguyen D.D., Lai J.-Y. (2022). Synthesis, bioactive properties, and biomedical applications of intrinsically therapeutic nanoparticles for disease treatment. Chem. Eng. J..

[3] Ramamurthy C., Sampath K., Arunkumar P., Kumar M.S., Sujatha V., Premkumar K., Thirunavukkarasu C. (2013). Green synthesis and characterization of selenium nanoparticles and its augmented cytotoxicity with doxorubicin on cancer cells. Bioprocess Biosyst. Eng..

[4] Alhumaydhi F.A. (2022). Green Synthesis of Gold Nanoparticles Using Extract of Pistacia chinensis and Their In Vitro and In Vivo Biological Activities. J. Nanomater..

[5] Arya A., Chundawat T.S. (2020). Metal nanoparticles from algae: A green approach for the synthesis, characterization and their biological activity. Nanosci. Nanotechnol. Asia.

[6] Javed R., Zia M., Naz S., Aisida S.O., Ao Q. (2020). Role of capping agents in the application of nanoparticles in biomedicine and environmental remediation: Recent trends and future prospects. J. Nanobiotechnol..

[7] Ji Y., Thomas C., Tulin N., Lodhi N., Boamah E., Kolenko V., Tulin A.V. (2016). Charon mediates immune deficiency–driven PARP-1–dependent immune responses in *Drosophila*. J. Immunol..

[8] Nii-Trebi N.I. (2017). Emerging and neglected infectious diseases: Insights, advances, and challenges. BioMed Res. Int..

[9] Tong C., Zou W., Ning W., Fan J., Li L., Liu B., Liu X. (2018). Synthesis of DNA-guided silver nanoparticles on a graphene oxide surface: Enhancing the antibacterial effect and the wound healing activity. RSC Adv..

[10] Ahamed M., AlSalhi M.S., Siddiqui M. (2010). Silver nanoparticle applications and human health. Clin. Chim. Acta.

[11] Das C.A., Kumar V.G., Dhas T.S., Karthick V., Govindaraju K., Joselin J.M., Baalamurugan J. (2020). Antibacterial activity of silver nanoparticles (biosynthesis): A short review on recent advances. Biocatal. Agric. Biotechnol..

[12] Sibbald R.G., Contreras-Ruiz J., Coutts P., Fierheller M., Rothman A., Woo K. (2007). Bacteriology, inflammation, and healing: A study of nanocrystalline silver dressings in chronic venous leg ulcers. Adv. Ski. Wound Care.

[13] Liu X., Lee P.y., Ho C.m., Lui V.C., Chen Y., Che C.m., Tam P.K., Wong K.K. (2010). Silver nanoparticles mediate differential responses in keratinocytes and fibroblasts during skin wound healing. ChemMedChem.

[14] Shahverdi A.R., Fakhimi A., Shahverdi H.R., Minaian S. (2007). Synthesis and effect of silver nanoparticles on the antibacterial activity of different antibiotics against *Staphylococcus aureus* and *Escherichia coli*. Nanomed. Nanotechnol. Biol. Med..

[15] Newsholme P., Cruzat V.F., Keane K.N., Carlessi R., de Bittencourt P.I.H. (2016). Molecular mechanisms of ROS production and oxidative stress in diabetes. Biochem. J..

[16] McGarry T., Biniecka M., Veale D.J., Fearon U. (2018). Hypoxia, oxidative stress and inflammation. Free Radic. Biol. Med..

[17] Hayes J.D., Dinkova-Kostova A.T., Tew K.D. (2020). Oxidative stress in cancer. Cancer Cell.

[18] Butterfield D.A., Halliwell B. (2019). Oxidative stress, dysfunctional glucose metabolism and Alzheimer disease. Nat. Rev. Neurosci..

[19] Münzel T., Camici G.G., Maack C., Bonetti N.R., Fuster V., Kovacic J.C. (2017). Impact of oxidative stress on the heart and vasculature: Part 2 of a 3-part series. J. Am. Coll. Cardiol..

[20] Zulfiqar H., Amjad M.S., Mehmood A., Mustafa G., Binish Z., Khan S., Arshad H., Proćków J., Pérez de la Lastra J.M. (2022). Antibacterial, Antioxidant, and Phytotoxic Potential of Phytosynthesized Silver Nanoparticles Using Elaeagnus umbellata Fruit Extract. Molecules.

[21] Nagaich U., Gulati N., Chauhan S. (2016). Antioxidant and antibacterial potential of silver nanoparticles: Biogenic synthesis utilizing apple extract. J. Pharm..

[22] Ahmed M.J., Murtaza G., Rashid F., Iqbal J. (2019). Eco-friendly green synthesis of silver nanoparticles and their potential applications as antioxidant and anticancer agents. Drug Dev. Ind. Pharm..

[23] Hussain Z., Abourehab M.A., Khan S., Thu H.E. (2020). Silver nanoparticles: A promising nanoplatform for targeted delivery of therapeutics and optimized therapeutic efficacy. Metal Nanoparticles for Drug Delivery and Diagnostic Applications.

[24] Tehri N., Vashishth A., Gahlaut A., Hooda V. (2022). Biosynthesis, antimicrobial spectra and applications of silver nanoparticles: Current progress and future prospects. Inorg. Nano-Met. Chem..

[25] Fibrich B., Gao X., Puri A., Banga A.K., Lall N. (2020). In vitro antioxidant, anti-inflammatory and skin permeation of myrsine africana and its isolated compound myrsinoside B. Front. Pharmacol..

[26] Zhang L., Wei Y., Wang H., Wu F., Zhao Y., Liu X., Wu H., Wang L., Su H. (2020). Green synthesis of silver nanoparticles using mushroom *Flammulina velutipes* Extract and their antibacterial activity against aquatic pathogens. Food Bioprocess Technol..

[27] Juibari M.M., Abbasalizadeh S., Jouzani G.S., Noruzi M. (2011). Intensified biosynthesis of silver nanoparticles using a native extremophilic *Ureibacillus thermosphaericus* strain. Mater. Lett..

[28] Narayanan K.B., Park H.H. (2014). Antifungal activity of silver nanoparticles synthesized using turnip leaf extract (*Brassica rapa* L.) against wood rotting pathogens. Eur. J. Plant Pathol..

[29] Fouda A., Eid A.M., Abdel-Rahman M.A., El-Belely E.F., Awad M.A., Hassan S.E.-D., Al-Faifi Z.E., Hamza M.F. (2022). Enhanced antimicrobial, cytotoxicity, larvicidal, and repellence activities of brown algae, cystoseira crinita-mediated green synthesis of magnesium oxide nanoparticles. Front. Bioeng. Biotechnol..

[30] Khan F.A., Zahoor M., Jalal A., Rahman A.U. (2016). Green synthesis of silver nanoparticles by using *Ziziphus nummularia* leaves aqueous extract and their biological activities. J. Nanomater..

[31] Anandalakshmi K., Venugobal J., Ramasamy V. (2016). Characterization of silver nanoparticles by green synthesis method using *Pedalium murex* leaf extract and their antibacterial activity. Appl. Nanosci..

[32] Wang D., Xue B., Wang L., Zhang Y., Liu L., Zhou Y. (2021). Fungus-mediated green synthesis of nano-silver using *Aspergillus sydowii* and its antifungal/antiproliferative activities. Sci. Rep..

[33] Valgas C., Souza S.M.D., Smânia E.F., Smânia A. (2007). Screening methods to determine antibacterial activity of natural products. Braz. J. Microbiol..

[34] Molyneux P. (2004). The use of the stable free radical diphenylpicrylhydrazyl (DPPH) for estimating antioxidant activity. Songklanakarin J. Sci. Technol..

[35] Vanaja M., Gnanajobitha G., Paulkumar K., Rajeshkumar S., Malarkodi C., Annadurai G. (2013). Phytosynthesis of silver nanoparticles by *Cissus quadrangularis*: Influence of physicochemical factors. J. Nanostruct. Chem..

[36] Salem S.S., El-Belely E.F., Niedbała G., Alnoman M.M., Hassan S.E.-D., Eid A.M., Shaheen T.I., Elkelish A., Fouda A. (2020). Bactericidal and in-vitro cytotoxic efficacy of silver nanoparticles (Ag-NPs) fabricated by endophytic actinomycetes and their use as coating for the textile fabrics. Nanomaterials.

[37] Kang J.H., Chae J.B., Kim C. (2018). A multi-functional chemosensor for highly selective ratiometric fluorescent detection of silver (I) ion and dual turn-on fluorescent and colorimetric detection of sulfide. R. Soc. Open Sci..

[38] Elumalai K., Velmurugan S. (2015). Green synthesis, characterization and antimicrobial activities of zinc oxide nanoparticles from the leaf extract of *Azadirachta indica* (L.). Appl. Surf. Sci..

[39] Alqadi M., Abo Noqtah O., Alzoubi F., Alzouby J., Aljarrah K. (2014). PH effect on the aggregation of silver nanoparticles synthesized by chemical reduction. Mater. Sci. Pol..

[40] Elemike E.E., Onwudiwe D.C., Ekennia A.C., Ehiri R.C., Nnaji N.J. (2017). Phytosynthesis of silver nanoparticles using aqueous leaf extracts of *Lippia citriodora*: Antimicrobial, larvicidal and photocatalytic evaluations. Mater. Sci. Eng. C.

[41] Loo Y.Y., Rukayadi Y., Nor-Khaizura M.-A.-R., Kuan C.H., Chieng B.W., Nishibuchi M., Radu S. (2018). In vitro antimicrobial activity of green synthesized silver nanoparticles against selected gram-negative foodborne pathogens. Front. Microbiol..

[42] Elmusa F., Aygun A., Gulbagca F., Seyrankaya A., Göl F., Yenikaya C., Sen F. (2021). Investigation of the antibacterial properties of silver nanoparticles synthesized using *Abelmoschus esculentus* extract and their ceramic applications. Int. J. Environ. Sci. Technol..

[43] Saravanan M., Barik S.K., MubarakAli D., Prakash P., Pugazhendhi A. (2018). Synthesis of silver nanoparticles from *Bacillus brevis* (NCIM 2533) and their antibacterial activity against pathogenic bacteria. Microb. Pathog..

[44] Mo F., Li H., Li Y., Chen X., Wang M., Li Z., Deng N., Yang Y., Huang X., Zhang R. (2021). Physiological, biochemical, and transcriptional regulation in a leguminous forage *Trifolium pratense* L. responding to silver ions. Plant Physiol. Biochem..

[45] Nimse S.B., Pal D. (2015). Free radicals, natural antioxidants, and their reaction mechanisms. RSC Adv..

[46] Abdel-Aziz M.S., Shaheen M.S., El-Nekeety A.A., Abdel-Wahhab M.A. (2014). Antioxidant and antibacterial activity of silver nanoparticles biosynthesized using *Chenopodium murale* leaf extract. J. Saudi Chem. Soc..

[47] Nayak S.P., Ramamurthy S.S., Kumar J.K. (2020). Green synthesis of silver nanoparticles decorated reduced graphene oxide nanocomposite as an electrocatalytic platform for the simultaneous detection of dopamine and uric acid. Mater. Chem. Phys..

[48] Khan G.A., Bouraine S., Wege S., Li Y., de Carbonnel M., Berthomieu P., Poirier Y., Rouached H. (2014). Coordination between zinc and phosphate homeostasis involves the transcription factor PHR1, the phosphate exporter PHO1, and its homologue PHO1; H3 in *Arabidopsis*. J. Exp. Bot..

[49] Goncharova N., Isamukhamedov A.S., Glushenkova A. (1993). Glycolipids and phospholipids of the fruit of *Elaeagnus angustifolia*. Chem. Nat. Compd..

[50] Yin L., Cheng Y., Espinasse B., Colman B.P., Auffan M., Wiesner M., Rose J., Liu J., Bernhardt E.S. (2011). More than the ions: The effects of silver nanoparticles on *Lolium multiflorum*. Environ. Sci. Technol..

[51] Pourmorad F., Hosseinimehr S., Shahabimajd N. (2006). Antioxidant activity, phenol and flavonoid contents of some selected Iranian medicinal plants. Afr. J. Biotechnol..

[52] Gardea-Torresdey J.L., Rico C.M., White J.C. (2014). Trophic transfer, transformation, and impact of engineered nanomaterials in terrestrial environments. Environ. Sci. Technol..

[53] Mirzajani F., Askari H., Hamzelou S., Farzaneh M., Ghassempour A. (2013). Effect of silver nanoparticles on *Oryza sativa* L. and its rhizosphere bacteria. Ecotoxicol. Environ. Saf..

[54] Thuesombat P., Hannongbua S., Akasit S., Chadchawan S. (2014). Effect of silver nanoparticles on rice (*Oryza sativa* L. cv. KDML 105) seed germination and seedling growth. Ecotoxicol. Environ. Saf..

[55] Mustafa G., Hasan M., Yamaguchi H., Hitachi K., Tsuchida K., Komatsu S. (2020). A comparative proteomic analysis of engineered and bio synthesized silver nanoparticles on soybean seedlings. J. Proteom..

[56] Hasan M., Mehmood K., Mustafa G., Zafar A., Tariq T., Hassan S.G., Loomba S., Zia M., Mazher A., Mahmood N. (2021). Phytotoxic evaluation of phytosynthesized silver nanoparticles on lettuce. Coatings.

[57] Mustafa G., Sakata K., Komatsu S. (2016). Proteomic analysis of soybean root exposed to varying sizes of silver nanoparticles under flooding stress. J. Proteom..

