# Correction: Sarwer et al. Green Synthesis and Characterization of Silver Nanoparticles Using *Myrsine africana* Leaf Extract for Their Antibacterial, Antioxidant and Phytotoxic Activities. *Molecules* 2022, *27*, 7612

**DOI:** 10.3390/molecules29091922

**Published:** 2024-04-23

**Authors:** Qudsia Sarwer, Muhammad Shoaib Amjad, Ansar Mehmood, Zakia Binish, Ghazala Mustafa, Atikah Farooq, Mirza Faisal Qaseem, Fozia Abasi, José Manuel Pérez de la Lastra

**Affiliations:** 1 Department of Botany, Women University of Azad Jammu & Kashmir, Bagh 12500, Pakistan; 2 School of Geography, Earth and Environmental Sciences, University of Birmingham, Birmingham B15 2TT, UK; 3 Department of Botany, University of Poonch, Rawlakot 12350, Pakistan; 4 Department of Plant Sciences, Quaid-i-Azam University, Islamabad 45320, Pakistan; 5 Department of Environmental Science and Forestry, Connecticut Agricultural Experiment Station, 123 Huntington Street, New Haven, CT 06511, USA; 6 Department of Botany, PMAS-University of Arid Agriculture, Rawalpindi 44000, Pakistan; 7 Biotecnología de Macromoléculas, Instituto de Productos Naturales y Agrobiología, (IPNA-CSIC), 38206 San Cristóbal de la Laguna, Spain

The authors wish to make the following corrections to this paper [1]. The authors state that the scientific conclusions are unaffected. This correction was approved by the Academic Editor. The original publication has also been updated.

2. Material and Methods
2.1. Collection of Sample and Preparation of Plant Extract

In the original publication, some information about the plant material and the collection date was missing. The correct information appears below.

The leaves of *M. africana* were collected by Qudsia Sarwer from Kahutta Azad Jammu and Kashmir in April 2021. The plant was identified by Dr. Muhammad Shoaib Amjad with the help of Flora of Pakistan and a voucher specimen (voucher number 278) was deposited in the Herbarium of the Department of Botany, Women University of Azad Jammu and Kashmir Bagh (Section 2.1).

2.4. Biological Activities
2.4.1. Antibacterial Activity

In the original publication, the information of the number of bacterial strains is incorrect.

The correct information appears below.

Four putative bacterial pathogens, *Pseudomonas aeruginosa*, *Escherichia coli*, *Staphylococcus aurous* and *Klebsiella pneumoniae*, were used for antibacterial activity.

Figure 1

In the original publication, the reference to the origin of the *Myrsine africana* picture was missing. It is now cited in the figure legend as follows:

The image of *M. africana* used in this figure can be found on Wikipedia (https://en.wikipedia.org/wiki/Myrsine_africana), last accessed on 17 September 2022.

Funding

Some information was missing from the funding section. It has now been added as follows:

No funds were exchanged between the collaborating countries.

Acknowledgments

Some information was missing from the acknowledgement section. It has now been added as follows:

We are also thankful to the National Institute of Laser and Optronics (NILOP) and the University of Azad Jammu & Kashmir Muzaffarabad for providing characterization facilities for nanoparticles.
